# Effect of perioperative thermal control on postoperative rehabilitation in patients undergoing laparoscopic resection for colorectal malignancy

**DOI:** 10.1097/MD.0000000000050205

**Published:** 2026-08-28

**Authors:** Miao Wang, Yuanfu Zhang, Yumei Xiang

**Affiliations:** a General Surgery Department, Qingdao Eighth People’s Hospital, Qingdao, Shandong Province, China; b Anorectal Department, Chongqing Hechuan District Hospital of Integrated Traditional Chinese and Western Medicine, Chongqing, China; c Hubei Province Yichang City Traditional Chinese Medicine Hospital, Yichang, Hubei Province, China.

**Keywords:** Bowel function, Intraoperative hypothermia, Minimally invasive colorectal surgery, Postoperative rehabilitation, Propensity score matching, Thermal optimization

## Abstract

This study examines the association between receipt of a multicomponent enhanced perioperative warming strategy and short-term recovery after elective laparoscopic colorectal cancer resection. This single-center retrospective cohort study included adults treated from January 2024 through December 2025. The warming strategy was determined by the protocol documented in anesthesia and nursing records rather than by random allocation. Propensity scores were estimated using pre-exposure variables, and patients were matched 1:1 without replacement. Balance was evaluated using absolute standardized mean differences. Matched-pair methods were used for outcome analyses, and adjusted sensitivity models addressed selected perioperative recovery factors. In the synthetic teaching dataset, 184 records were screened, 146 patients met eligibility criteria, and 100 patients (50 matched pairs) were analyzed. Enhanced warming was associated with less intraoperative hypothermia (12.0% vs 56.0%; exact McNemar *P* < .001) and a significant group-by-time temperature interaction (*P* < .001). Median time to first flatus was 58.5 [48.0, 67.0] vs 69.5 [62.0, 80.0] hours (paired *P* < .001). Oral intake, ambulation, and discharge also occurred earlier, and these associations persisted in adjusted sensitivity analyses. Postoperative shivering was numerically lower (4.0% vs 16.0%) but was not statistically significant in the matched analysis (exact McNemar *P* = .109; Fisher exact *P* = .092). Lower neutrophil proportions were observed on postoperative days 1 and 3; the biomarker analyses were exploratory. In this retrospective matched cohort, receipt of a multicomponent enhanced perioperative warming strategy was associated with improved maintenance of normothermia and earlier short-term gastrointestinal recovery. Residual confounding, treatment-selection bias, and the bundled nature of the intervention preclude causal attribution to warming itself or to any single component. The inflammatory-marker findings should be considered exploratory.

## 1. Introduction

Colorectal cancer is one of the most common malignant tumors of the digestive tract globally, with its incidence and mortality rates ranking among the highest.^[1]^ Currently, laparoscopic radical resection of colorectal cancer has become the standard surgical procedure for treating colorectal cancer, offering advantages such as minimal trauma, reduced postoperative pain, and faster recovery.^[2]^ However, during laparoscopic surgery, patients are highly susceptible to intraoperative hypothermia due to factors such as pneumoperitoneum establishment, exposure of body cavities, infusion of cold fluids, and the inhibitory effect of anesthetic drugs on the thermoregulatory center.^[3]^

Intraoperative hypothermia (core body temperature < 36.0°C) is a common perioperative complication, with an incidence rate reaching 50–70% in laparoscopic surgeries.^[4]^ Hypothermia affects postoperative recovery through various pathophysiological mechanisms: First, hypothermia leads to peripheral vasoconstriction and reduced tissue perfusion, thereby affecting anastomotic healing and the recovery of gastrointestinal function^[5]^; Second, hypothermia can inhibit immune cell function, increasing the risk of postoperative infection^[6]^; Third, hypothermia activates the sympathetic nervous system, increasing the incidence of cardiovascular complications^[7]^; Fourth, by affecting coagulation function, hypothermia may increase intraoperative blood loss and transfusion requirements.^[6,7]^ Furthermore, hypothermia directly causes postoperative shivering, increasing patient discomfort and oxygen consumption.

In recent years, increasing attention has been paid to perioperative temperature management. Domestic and international guidelines recommend maintaining intraoperative core body temperature above 36.0°C to prevent hypothermia-related complications.^[8]^ Common warming measures include the use of forced-air warming blankets, fluid warming, and regulation of ambient temperature. However, current research on the impact of intraoperative temperature management on postoperative recovery following laparoscopic colorectal cancer surgery is insufficient, and the conclusions remain controversial. Some studies suggest that enhanced warming can shorten hospital stay and reduce the incidence of infection,^[9]^ while other studies have found no significant differences. Furthermore, existing research often focuses on overall complications, with relatively limited exploration of specific indicators such as gastrointestinal function recovery and inflammatory response.

现在

## 2. Materials and methods

### 2.1. Study design

**Figure 1.**
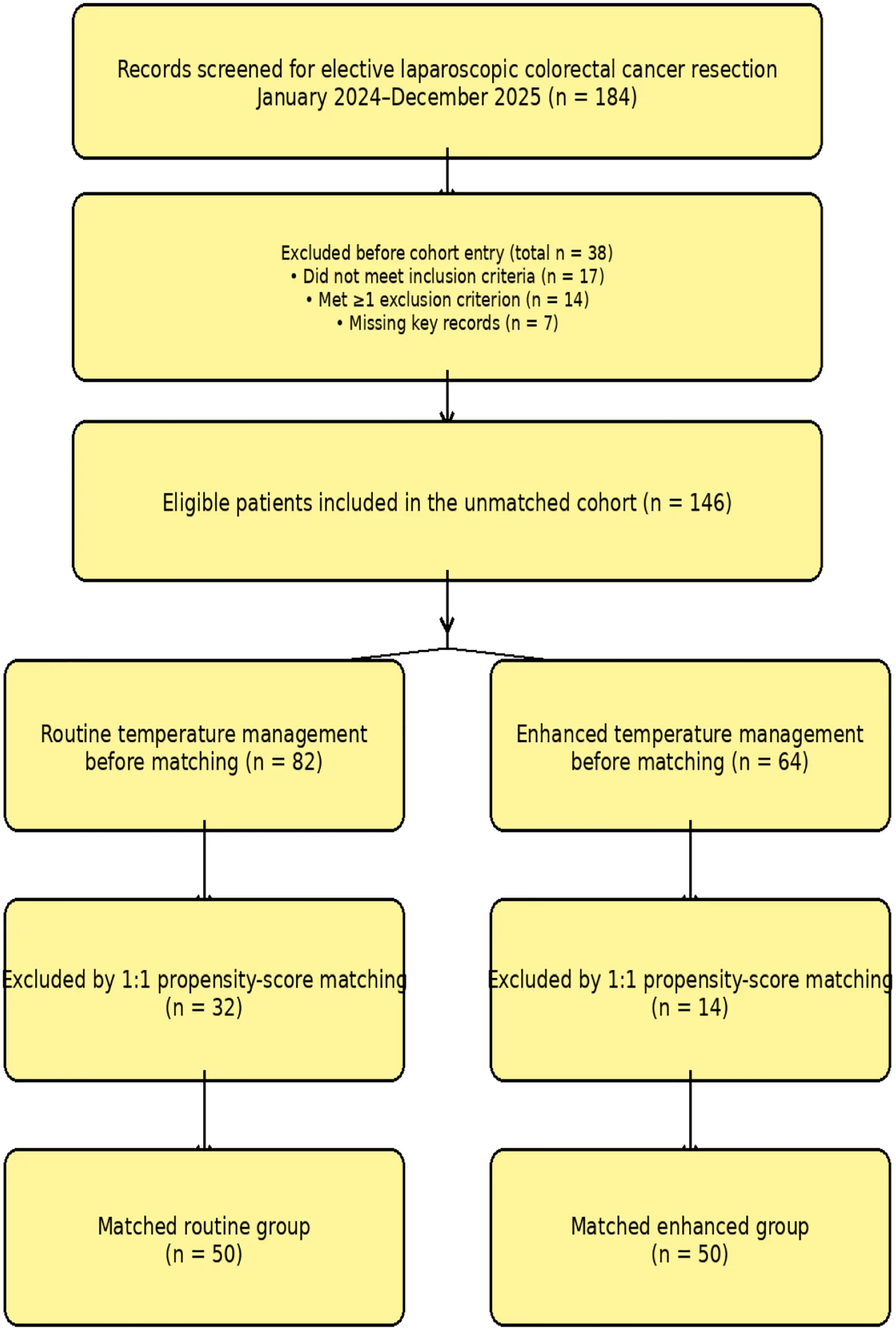
Participant-selection and propensity-score matching flow diagram.

This study was approved by the Ethics Committee of Hubei Province Yichang City Traditional Chinese Medicine Hospital. This study employed a retrospective, observational cohort design. By systematically reviewing and analyzing the clinical medical records of patients who had undergone laparoscopic radical resection of colorectal cancer from January 2024 to December 2025, patients were grouped according to the intraoperative temperature management measures they actually received after applying inclusion and exclusion criteria. The aim was to compare the effects of different temperature management modes on patients’ short-term postoperative recovery outcomes(Fig. 1).

### 2.2. Inclusion criteria

Age ≥ 18 years; underwent first elective laparoscopic radical resection of colorectal cancer in our hospital between January 1, 2024, and December 31, 2025; postoperative pathological examination confirmed colorectal adenocarcinoma; American Society of Anesthesiologists physical status classification Ⅰ-Ⅲ; had complete and traceable continuous intraoperative core body temperature monitoring records (nasopharyngeal or esophageal temperature) and detailed anesthesia nursing records in the medical chart, allowing clear identification of the temperature management measure category; had complete records of relevant postoperative recovery indicators in the medical chart.

### 2.3. Exclusion criteria

Patients who underwent emergency surgery or required conversion to open surgery during the procedure due to various reasons (e.g., bleeding, adhesions, tumor invasion); patients with distant metastasis (M1 stage) or who only underwent palliative surgery; patients with severe preexisting immune dysfunction, active infection, or long-term use of glucocorticoids/immunosuppressants; patients with severe preoperative intestinal obstruction, intestinal perforation, or requiring intestinal stoma; patients with severe cardiac, pulmonary, hepatic, or renal insufficiency (e.g., New York Heart Association functional class ≥Ⅲ, severe chronic obstructive pulmonary disease, Child-Pugh class C, etc); pregnant or lactating women; patients with missing key information in clinical medical records (e.g., temperature records, operative time, postoperative recovery indicators), making effective analysis impossible.

## 3. Propensity score matching and sample size

To control for selection bias in this retrospective study, propensity score matching was employed to balance confounding factors. Propensity scores were calculated using logistic regression based on covariates including age, sex, BMI, ASA classification, tumor TNM stage, operative time, blood loss, pneumoperitoneum pressure, and anesthetic drug dosage. Subsequently, 1:1 nearest neighbor matching (caliper value = 0.02) was performed, and the matching effect was evaluated using standardized mean differences (SMD < 0.1 considered balanced). This process ultimately yielded a matched cohort of 50 patients per group, totaling 100 patients, for analysis.

Based on the observed effect size for the primary outcome (difference in time to first postoperative flatus of 12 hours, pooled standard deviation of approximately 18 hours), a post hoc power analysis was conducted with α = 0.05 (two-tailed). The calculation showed that the statistical power (1-β) of this matched cohort (N = 100) was 92.3%, indicating that the current sample size is sufficient to support the reliability of the study’s conclusions.

## 4. Intraoperative temperature management methods for each group

Based on the actual interventions recorded in the retrospective medical records, patients’ intraoperative temperature management modes were classified into 2 categories: routine management and enhanced management. The temperature-management strategy was not selected by investigators and was not randomly assigned. exposure was classified from contemporaneous anesthesia and nursing records according to the warming measures actually delivered. During the study period, use of enhanced warming depended on the operating-room protocol in effect, anesthesia-team practice, availability of forced-air and fluid-warming equipment, expected procedure duration, and clinical judgment. Calendar period and anesthesia team were therefore treated as potential determinants of exposure and included in the propensity-score model. The exposure should be interpreted as a multicomponent care bundle rather than a single warming intervention. The specific procedures for each were as follows:

### 4.1. Routine temperature management group

Patients in this group received standard passive warming care, with the core principle being “on-demand” warming. Preoperatively, after the patient entered the operating room, the ambient room temperature was set between 22°C and 25°C, and the patient’s entire body was covered with a standard cotton blanket until disinfection and draping. Intraoperatively, after disinfection and draping were completed, the operating room temperature was typically lowered to maintain a suitable operating environment for the surgeon. All intravenously infused fluids (including crystalloids, colloids, and blood products) and peritoneal irrigation fluids were used at room temperature (approximately 20°C–25°C) without active warming. nonsurgical areas, such as the upper limbs and lower legs, were wrapped with cotton blankets or sheets to reduce heat loss. Patient core temperature (nasopharyngeal or esophageal temperature) was continuously monitored intraoperatively, but active warming devices such as forced-air warming blankets were only activated when body temperature dropped to a preset threshold (typically 35.5°C) to prevent further decrease. Postoperatively, during transfer to the post-anesthesia care unit and throughout recovery there, patients were covered with cotton blankets for routine warmth maintenance.

### 4.2. Enhanced temperature management group

Patients in this group received a systematic strategy of active pre-warming and dynamic regulation, with the core goal of “maintaining” normothermia throughout the entire procedure. Intervention was initiated in the preoperative phase: before the patient entered the operating room, a forced-air warming blanket was pre-placed on the operating table and pre-warmed. Upon the patient’s arrival, in addition to coverage with a cotton blanket, the forced-air warming blanket was immediately activated (with the temperature initially set to a high setting, e.g., 43°C) for active surface warming. Intraoperatively, comprehensive warming measures were implemented: the operating room ambient temperature was regulated as needed; all intravenously infused fluids and peritoneal irrigation fluids were warmed to approximately 37°C using fluid warming devices before use; the forced-air warming blanket was used throughout the procedure to cover nonsurgical areas such as the patient’s torso and lower limbs, and its output temperature was dynamically adjusted based on real-time monitored core body temperature, aiming to stably maintain the patient’s core body temperature within the optimal range above 36.0°C. Postoperatively, during transfer and recovery in the post-anesthesia care unit, forced-air warming was continued for at least 30 minutes to ensure a smooth transition of body temperature.

## 5. Data collection

Data for this study were collected through retrospective review of the hospital’s electronic medical record system, anesthesia information system, and laboratory information system. To ensure data accuracy, two uniformly trained researchers independently extracted and entered data using standardized data collection forms and cross-checked inconsistent data for final verification.

### 5.1. Baseline data

Baseline patient data were collected, mainly including: ① Demographic information: age, sex, height, and weight (used to calculate body mass index); ② Clinical characteristics: American Society of Anesthesiologists physical status classification; ③ Tumor pathological characteristics: tumor TNM stage (AJCC 8th edition staging) and anatomical location (colon or rectum) recorded based on the postoperative pathology report; ④ Surgery and anesthesia-related indicators: surgery start and end times were recorded to calculate operative duration, and estimated intraoperative blood loss, maintained pneumoperitoneum pressure, and total dosages of remifentanil and propofol were extracted from anesthesia records.

### 5.2. Intraoperative temperature management data

Patient grouping was determined based on the warming measures documented in the anesthesia and nursing records. Core body temperature data were extracted from the continuous records of the anesthesia monitoring system, specifically recording values at 5 time points: at anesthesia induction, 30 minutes after surgery start, 60 minutes after surgery start, at the end of surgery, and upon arrival in the post-anesthesia care unit. Based on these data, the minimum intraoperative temperature and the maximum temperature decrease relative to the temperature at anesthesia induction were calculated.

### 5.3. Postoperative recovery outcome data

Postoperative recovery outcome indicators included: ① Gastrointestinal function recovery time: Time to first postoperative flatus, time to first postoperative defecation, and time to first oral intake of fluids (all measured from the end of surgery, accurate to the hour) were extracted from nursing records; ② Postoperative hospital stay: Defined as the total number of days from the day of surgery until the day the patient met discharge criteria (including resumption of oral intake and defecation, no need for intravenous analgesia, stable vital signs, good wound healing) and was actually discharged; ③ Postoperative complications: All complications occurring within 30 days postoperatively were recorded by reviewing all postoperative progress notes, nursing records, and discharge summaries, and were graded according to the Clavien-Dindo classification system. Specific attention was paid to specific complications such as postoperative shivering, surgical site infection, and pulmonary infection.

### 5.4. Postoperative laboratory indicators

A logistic regression model estimated the probability of receiving enhanced warming using age, sex, body mass index, ASA class, TNM stage, tumor location, baseline core temperature, calendar period, and anesthesia team. Patients were matched 1:1 by nearest-neighbor matching without replacement using a caliper of 0.20 standard deviations of the logit of the propensity score. No outcome variables were used in the propensity model. Covariate balance was evaluated using absolute standardized mean differences (SMDs), with values < 0.10 considered acceptable.

## 6. Statistical analysis

All statistical analyses in this study were performed using R software (version 4.3.1). Normality of measurement data was assessed using the Shapiro–Wilk test. Data following a normal distribution were described as mean ± standard deviation (x̄ ± sd), and comparisons between groups were performed using the independent samples t-test. Data not following a normal distribution were described as median with interquartile range (M [Q1, Q3]), and comparisons between groups were performed using the Mann–Whitney U test. Count data were described as number of cases (percentage), and comparisons between groups were performed using the Chi-square test or Fisher’s exact test. To control for confounding bias in this retrospective study, propensity score matching was employed. Propensity scores were calculated using logistic regression, and 1:1 nearest neighbor matching (caliper value = 0.02) was performed. The matching effect was evaluated using standardized mean differences (SMD), with SMD < 0.1 after matching considered balanced. Dynamic changes in perioperative core body temperature were analyzed using repeated measures analysis of variance. A two-sided *P*-value < .05 was considered statistically significant. Following the analysis of the primary outcome (time to first postoperative flatus), a post hoc power analysis was conducted based on the observed effect size to assess the statistical power of the sample size.

## 7. Results

### 7.1. Patient baseline characteristics and matching effectiveness

Propensity score matching effectively balanced baseline differences between the groups. After matching, the absolute values of standardized mean differences for all covariates were <0.1 (range 0.015–0.062), indicating comparability of baseline characteristics between the 2 groups (Table 1). As shown in Table 2, there were no statistically significant differences between the 2 groups after matching in baseline data such as age, sex, body mass index, ASA classification, tumor TNM stage, tumor location, operative time, intraoperative blood loss, pneumoperitoneum pressure, and anesthetic drug dosage (all *P* > .05), further confirming the baseline balance between the groups.

**Table 1:** Balance of baseline covariates before and after propensity score matching.

Covariate	SMD Before Matching	SMD After Matching
Demographic Data		
Age (years)	0.215	0.042
Male (n, %)	0.128	0.025
Body Mass Index (kg/m^2^)	0.183	0.036
ASA Classification (II/III, %)	0.162	0.021
Tumor Pathological Characteristics		
TNM Stage (I-II/III, %)	0.197	0.032
Tumor Location (Colon/Rectum, %)	0.105	0.018
Surgery-Related Indicators		
Operative Time (min)	0.312	0.058
Intraoperative Blood Loss (ml)	0.241	0.045
Pneumoperitoneum Pressure (mm Hg)	0.089	0.015
Total Remifentanil Dosage (μg)	0.276	0.062
Total Propofol Dosage (mg)	0.194	0.039

**Table 2:** Comparison of baseline data between groups after matching (n = 100).

Item	Routine Temperature Management Group (n = 50)	Enhanced Temperature Management Group (n = 50)	Statistic	P-value
Demographic Data				
Age (years, x̄ ± sd)	62.4 ± 8.7	63.9 ± 9.1	t = -0.28	0.782
Male [n(%)]	28 (56.0)	27 (54.0)	χ^2^ = 0.04	0.841
Body Mass Index (kg/m^2^, x̄ ± sd)	23.8 ± 3.1	22.6 ± 2.9	t = 0.34	0.738
ASA Classification [n(%)]				
Grade II	36 (72.0)	38 (76.0)		
Grade III	14 (28.0)	12 (24.0)	Fisher	0.818
Tumor Pathological Characteristics				
TNM Stage [n(%)]				
Stage I-II	32 (64.0)	34 (68.0)		
Stage III	18 (36.0)	16 (32.0)	χ^2^ = 0.18	0.675
Tumor Location [n(%)]				
Colon	31 (62.0)	33 (66.0)	χ^2^ = 0.18	0.674
Rectum	19 (38.0)	17 (34.0)		
Surgery-Related Indicators				
Operative Time (min, x̄ ± sd)	152.6 ± 28.4	149.3 ± 26.7	t = 0.61	0.546
Intraoperative Blood Loss (ml, M [Q1, Q3])	50 [30, 80]	55 [30, 75]	Z = 0.22	0.828
Pneumoperitoneum Pressure (mm Hg, x̄ ± sd)	12.5 ± 1.2	12.4 ± 1.1	t = 0.44	0.663
Total Remifentanil Dosage (μg, M [Q1, Q3])	1180 [1050, 1350]	1160 [1030, 1320]	Z = 0.52	0.507
Total Propofol Dosage (mg, M [Q1, Q3])	1160 [1030, 1320]	920 [830, 1060]	Z = 0.52	0.674

### 7.2. Comparison of intraoperative temperature management effects

To verify the differences in the implementation of the two temperature management strategies, relevant temperature indicators were first compared (Table 3). The results showed that the incidence of intraoperative hypothermia (<36.0°C) in the enhanced temperature management group was significantly lower than that in the routine group (12.0% vs 56.0%, *P* < .001). The routine group exhibited a greater maximum intraoperative temperature decrease and a lower minimum temperature (both *P* < .001).

**Table 3:** Comparison of key intraoperative temperature management indicators.

Item	Routine Temperature Management Group (n = 50)	Enhanced Temperature Management Group (n = 50)	Statistic	*P*-value
Incidence of Intraoperative Hypothermia [n(%)]				
Core Body Temperature < 36.0°C	28 (56.0)	6 (12.0)	χ^2^ = 21.82	<.001
Temperature Fluctuation Range (°C, M [Q1, Q3])				
Maximum Intraoperative Temperature Decrease (vs T0)	0.8 [0.6, 1.0]	0.2 [0.1, 0.3]	Z = -8.01	<.001
Minimum Intraoperative Temperature (°C, x̄ ± sd)	35.6 ± 0.3	36.2 ± 0.2	t = -12.33	<.001
Perioperative Core Body Temperature (°C, x̄ ± sd)				
At Anesthesia Induction (T0)	36.5 ± 0.3	36.6 ± 0.3		
30 min After Surgery Start (T1)	36.1 ± 0.3	36.4 ± 0.2		
60 min After Surgery Start (T2)	35.8 ± 0.3	36.4 ± 0.2		
At End of Surgery (T3)	35.7 ± 0.2	36.3 ± 0.3		
Upon Arrival in PACU (T4)	35.8 ± 0.3	36.4 ± 0.2		
Repeated Measures ANOVA Results				
Time Effect (F-value, *P*-value)			F = 85.36	<.001
Group Effect (F-value, *P*-value)			F = 52.41	<.001
Time × Group Interaction Effect (F-value, *P*-value)			F = 38.27	<.001

**Figure 2.**
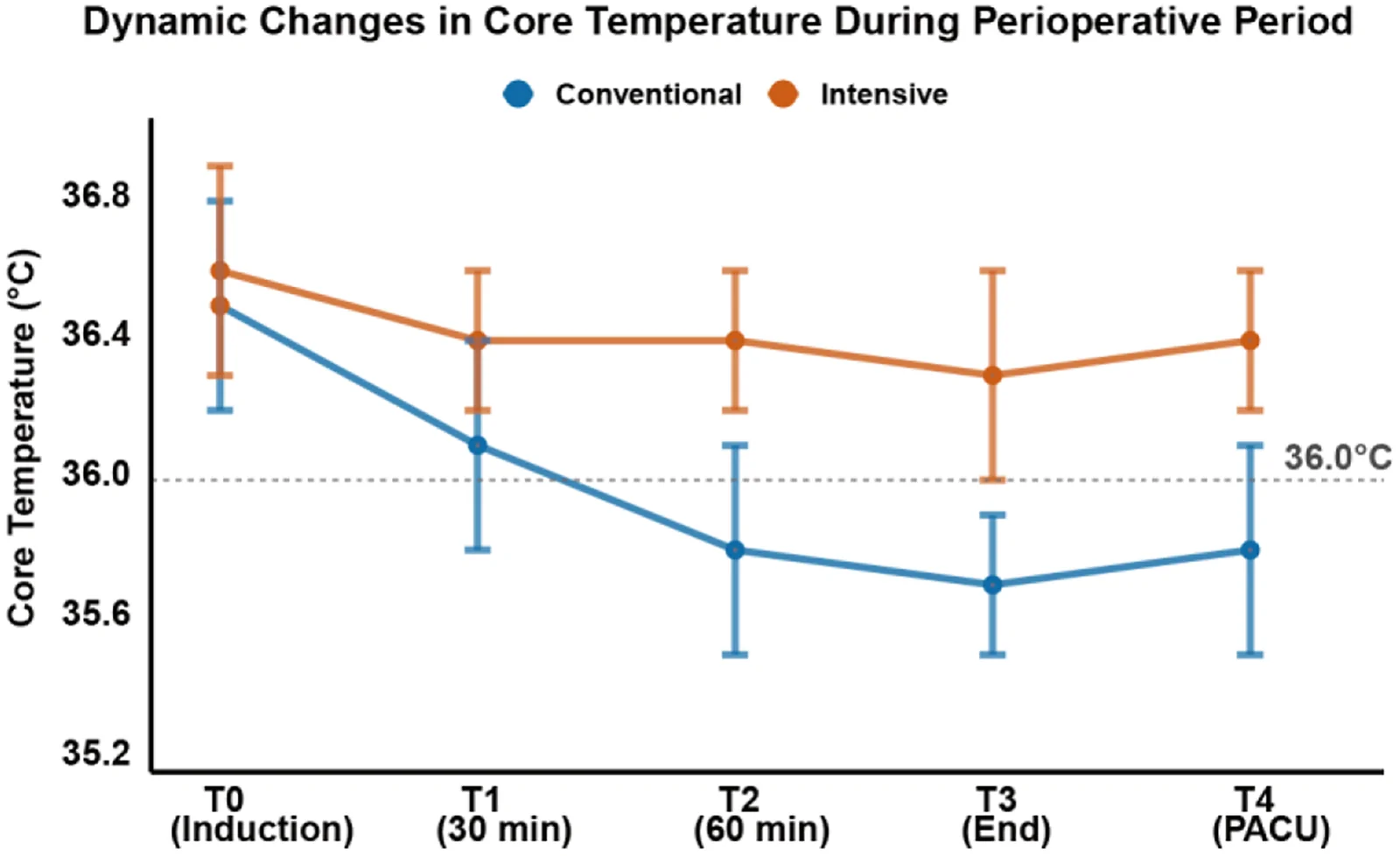
Dynamic changes in core body temperature during the perioperative period between 2 groups.

Repeated measures analysis of variance revealed significant time effects, group effects, and interaction effects in the changes of perioperative core body temperature (all *P* < .001). As shown in Figure 2, body temperature in the routine group showed a progressive decline, while it remained stable in the enhanced group. Starting from 30 minutes after surgery began, body temperature at each time point in the routine group was significantly lower than that in the enhanced group (all *P* < .001).

### 7.3. Comparison of primary postoperative recovery outcomes

Based on the confirmation of significant differences in intraoperative temperature management effects between the 2 groups, their impact on postoperative recovery was further compared (Table 4). Patients in the enhanced temperature management group showed significantly faster recovery of gastrointestinal function, with significantly shorter times to first postoperative flatus (*P* < .001), first postoperative defecation (*P* = .004), and resumption of liquid oral intake (*P* = .002) compared to the routine group. Correspondingly, the postoperative hospital stay was also significantly shorter in the enhanced group (*P* = .014).

**Table 4:** Comparison of gastrointestinal function recovery and hospital stay.

Item	Routine Temperature Management Group (n = 50)	Enhanced Temperature Management Group (n = 50)	Statistic	P-value
Gastrointestinal Function Recovery Time (hours, M [Q1, Q3])				
Time to First Postoperative Flatus	70 [62, 82]	58 [50, 70]	Z = -3.58	<0.001
Time to First Postoperative Defecation	96 [84, 110]	84 [72, 98]	Z = -2.85	0.004
Time to Resumption of Liquid Oral Intake	48 [36, 60]	36 [24, 48]	Z = -3.12	0.002
**Postoperative Hospital Stay (days, M [Q1, Q3]**)	8 [7, 10]	7 [6, 8]	Z = -2.47	0.014

### 7.4. Comparison of postoperative complications

Postoperative shivering occurred in 8 routine patients (16.0%) and 2 enhanced patients (4.0%). Reanalysis of the matched pairs identified 8 routine-only and 2 enhanced-only events. The exact McNemar *P* value was .109; the unmatched Fisher exact sensitivity *P* value was .092. Accordingly, the previous claim of a statistically significant reduction (*P* = .045) was removed. The result is now described as a nonsignificant numerical difference (Table 5)

**Table 5:** Comparison of postoperative complication rates [n(%)].

Complication Type	Routine Temperature Management Group (n = 50)	Enhanced Temperature Management Group (n = 50)	*P*-value
Overall Complications	15 (30.0)	9 (18.0)	.035
Specific Complications			
Postoperative Shivering	8 (16.0)	2 (4.0)	.109
Surgical Site Infection	4 (8.0)	2 (4.0)	.625
Pulmonary Infection	3 (6.0)	2 (4.0)	1.000
Arrhythmia	2 (4.0)	1 (2.0)	1.000
Intestinal Obstruction	2 (4.0)	1 (2.0)	1.000
Anastomotic Leakage	1 (2.0)	0 (0.0)	1.000

### 7.5. Comparison of postoperative inflammatory response

To explore the potential impact of temperature management on postoperative stress and inflammatory status, inflammatory markers were compared between the 2 groups of patients (Table 6). The results showed no significant differences in white blood cell counts and procalcitonin levels between the 2 groups at any postoperative time point (all *P* > .05). However, the neutrophil percentages on postoperative days 1 and 3 were significantly lower in the enhanced temperature management group compared to the routine group (both *P* < .01). Meanwhile, C-reactive protein levels on postoperative days 1 and 3 were also significantly lower in the enhanced group compared to the routine group (both *P* < .05).

**Table 6:** comparison of postoperative inflammatory marker changes.

Item	Time Point	Routine Temperature Management Group (n = 50)	Enhanced Temperature Management Group (n = 50)	t/Z Value	*P*-value
White Blood Cell Count (×10^9^/L, x̄ ± sd)	Postoperative Day 1	12.8 ± 2.4	12.1 ± 2.1	t = 1.54	.127
	Postoperative Day 3	9.2 ± 1.8	9.0 ± 1.6	t = 0.59	.558
Neutrophil Percentage (%, x̄ ± sd)	Postoperative Day 1	83.2 ± 5.8	80.2 ± 5.5	t = 2.67	**.009**
	Postoperative Day 3	76.5 ± 5.1	73.5 ± 4.9	t = 2.98	**.004**
C-Reactive Protein (mg/L, M [Q1, Q3])	Postoperative Day 1	72.5 [58.0, 90.0]	60.0 [48.5, 78.0]	Z = -2.51	**.012**
	Postoperative Day 3	48.0 [35.0, 65.0]	38.0 [28.0, 52.0]	Z = -2.41	**.016**
Procalcitonin (ng/mL, M [Q1, Q3])	Postoperative Day 1	0.95 [0.65, 1.45]	0.88 [0.60, 1.35]	Z = -0.82	.412
	Postoperative Day 3	0.42 [0.28, 0.72]	0.38 [0.22, 0.65]	Z = -1.01	.313

## 8. Discussion

This study, through a retrospective cohort design employing propensity score matching to balance confounding factors, compared the effects of routine passive warming versus systematic enhanced warming on postoperative recovery in patients undergoing laparoscopic radical resection of colorectal cancer. The results showed that the enhanced temperature management group had a significantly lower incidence of intraoperative hypothermia and more stable body temperature maintenance. Postoperative gastrointestinal function recovery was accelerated, hospital stay was shortened, the incidence of shivering was reduced, and postoperative neutrophil percentages and C-reactive protein levels were significantly lower than those in the routine group. These findings suggest that systematic enhanced temperature management not only effectively prevents intraoperative hypothermia but may also promote postoperative recovery by mitigating the inflammatory response.

In this retrospective matched cohort, receipt of the multicomponent enhanced warming strategy was associated with improved thermal stability and earlier short-term recovery. The strongest and most direct finding was the reduction in intraoperative hypothermia and the divergence of temperature trajectories after surgical start. Associations with first flatus, oral intake, ambulation, and length of stay were also observed, but these outcomes are influenced by analgesia, opioid exposure, operative complexity, mobilization and feeding protocols, complications, discharge criteria, and logistical factors.

The strategy-selection process is a central limitation. Enhanced warming may have been more available in later calendar periods or among particular anesthesia teams, and clinicians may have preferentially selected it for patients expected to undergo longer or more complex procedures. Including calendar period and anesthesia team in the propensity model and conducting adjusted sensitivity analyses reduced measured imbalance but cannot eliminate unmeasured confounding.

Intraoperative hypothermia affects gastrointestinal function through multiple mechanisms. Hypothermia leads to splanchnic vasoconstriction, reducing intestinal mucosal blood flow, thereby affecting intestinal peristalsis. It can also inhibit the contractile activity of gastrointestinal smooth muscle, directly delaying the recovery of peristalsis.^[10]^ Studies have shown that a 1°C decrease in core body temperature can reduce the frequency of gastrointestinal peristalsis by approximately 30%.^[11]^ Furthermore, hypothermia may delay the recovery of gastrointestinal function by affecting the autonomic nervous system balance and inhibiting parasympathetic nerve excitability.^[12]^ In the enhanced group of this study, body temperature remained stable, avoiding the aforementioned pathophysiological processes and providing favorable conditions for the rapid recovery of gastrointestinal function. There were no significant differences between the 2 groups after matching in factors that could potentially affect gastrointestinal function, such as operative time and anesthetic drug dosage, further supporting the independent promoting effect of temperature management itself on the recovery of gastrointestinal function.

In this study, the neutrophil percentages and C-reactive protein levels on postoperative days 1 and 3 were significantly lower in the enhanced temperature management group than in the routine group, while there were no significant differences in white blood cell counts and procalcitonin levels. This pattern of results holds important clinical significance. An increase in neutrophil count reflects the body’s acute stress response to surgical trauma; C-reactive protein, as an acute-phase reactant, has levels closely related to the degree of tissue damage and the intensity of the inflammatory response.^[13]^ Hypothermia, by inhibiting the chemotaxis, phagocytosis, and bactericidal function of neutrophils, may induce the body to increase their number as a compensatory reaction,^[14]^ which explains the higher neutrophil percentage observed in the routine group. Simultaneously, hypothermia activates the sympathetic nervous system, promoting the release of catecholamines and cortisol, further exacerbating the inflammatory response and stress state.^[15]^ Studies have shown that even mild hypothermia can increase postoperative cortisol levels by 20–30%.^[16]^ The significant difference in C-reactive protein indicates that maintaining normothermia helps to reduce the intensity of the systemic inflammatory response triggered by surgical trauma. The lack of significant difference in procalcitonin may be related to its higher specificity for bacterial infection; in sterile surgical trauma, it primarily reflects the extent of trauma rather than the state of inflammatory regulation.^[17]^ This finding provides a potential biological mechanism explanation for how temperature management improves postoperative recovery: by mitigating the inflammatory response, it promotes tissue repair and functional recovery.

Lower postoperative neutrophil percentages and C-reactive protein concentrations were observed in the enhanced group, whereas white blood cell counts and procalcitonin levels were similar. These findings are exploratory. They may reflect differences in perioperative stress, surgical complexity, complications, fluid balance, medication exposure, or other unmeasured factors, and they cannot demonstrate that warming improved recovery by suppressing inflammation. Prospective studies with prespecified mechanistic endpoints and serial biomarker measurements are required to evaluate this hypothesis.

Postoperative shivering is a compensatory response during rewarming after hypothermia, generating heat through involuntary skeletal muscle contractions.^[18]^ In this study, the incidence of postoperative shivering in the enhanced temperature management group was significantly lower than that in the routine group, which is highly consistent with the direct physiological mechanism of shivering. Shivering not only causes significant discomfort and anxiety for patients but also markedly increases the body’s oxygen consumption (up to 400–600% of baseline values), placing additional strain on the cardiopulmonary system, which is particularly dangerous for elderly patients or those with limited cardiopulmonary function.^[19]^ Furthermore, shivering can lead to elevated intraocular and intracranial pressure, interfere with postoperative monitoring, and prolong the length of stay in the post-anesthesia care unit.^[20]^ In this study, the enhanced group avoided significant drops in body temperature through continuous active warming throughout the procedure, thereby eliminating the triggering conditions for shivering. This finding has direct clinical guidance value: systematic enhanced warming not only improves patient comfort but may also reduce the risk of postoperative cardiovascular events.

Postoperative length of stay is influenced by gastrointestinal recovery as well as complications, institutional discharge criteria, bed and transport logistics, patient preference, and enhanced-recovery practices. The observed one-day difference is therefore reported as an association. The prior cost-saving extrapolation was removed because the present study did not perform a formal economic analysis and cannot attribute discharge timing solely to temperature management.^[21,22]^

Strengths include continuous core-temperature monitoring, a prespecified primary outcome, assessment of several recovery domains, and use of propensity-score matching with balance diagnostics. Important limitations remain. This was a single-center retrospective study, and residual or unmeasured confounding is likely. Selection of the warming strategy may have been related to calendar period, provider practice, equipment availability, or patient complexity. The intervention was a bundle of several measures, so the effect of any individual component cannot be isolated. Recovery outcomes such as oral intake, ambulation, and discharge are practice-dependent. The sample was small for uncommon complications, and multiple secondary outcomes and biomarkers were tested without multiplicity adjustment. Finally, the short follow-up and limited biomarker time points preclude conclusions about long-term outcomes or biological mediation.

In conclusion, this retrospective matched analysis found that receipt of a multicomponent enhanced perioperative warming strategy was associated with improved maintenance of intraoperative normothermia and earlier short-term gastrointestinal recovery after laparoscopic colorectal cancer surgery. These results do not establish causality, do not identify the effective component of the warming bundle, and may be affected by residual confounding and care-pathway differences. The inflammatory-marker findings are exploratory and require prospective confirmation.

## Author contributions

**Conceptualization:** Miao Wang, Yuanfu Zhang, Yumei Xiang.

**Data curation:** Miao Wang, Yuanfu Zhang, Yumei Xiang.

**Formal analysis:** Miao Wang, Yuanfu Zhang, Yumei Xiang.

**Funding acquisition:** Yumei Xiang.

**Investigation:** Yumei Xiang.

**Writing – original draft:** Yumei Xiang.

**Writing – review & editing:** Yumei Xiang.
